# Type-2 Diabetes as a Risk Factor for Severe COVID-19 Infection

**DOI:** 10.3390/microorganisms9061211

**Published:** 2021-06-03

**Authors:** Mahnaz Norouzi, Shaghayegh Norouzi, Alistaire Ruggiero, Mohammad S. Khan, Stephen Myers, Kylie Kavanagh, Ravichandra Vemuri

**Affiliations:** 1 Department of Genetics, Faculty of Sciences, Shahid Chamran University of Ahvaz, Ahvaz 61355, Iran; noroozi.mahnaz@gmail.com; 2 School of Health and Biomedical Sciences, Royal Melbourne Institute of Technology University, Melbourne, VIC 3083, Australia; 3 Department of Pathology, Wake Forest School of Medicine, Medical Center Boulevard, Winston-Salem, NC 27157, USA; adruggie@wakehealth.edu (A.R.); kkavanag@wakehealth.edu (K.K.); 4 Center for Precision Medicine, Wake Forest School of Medicine, Medical Center Boulevard, Winston-Salem, NC 27157, USA; mokhan@wakehealth.edu; 5 College of Health and Medicine, School of Health Sciences, University of Tasmania, Hobart, TAS 7005, Australia; stephen.myers@utas.edu.au

**Keywords:** diabetes, SARS-CoV-2, immune response, adipose tissue, glucose metabolism, vaccines

## Abstract

The current outbreak caused by severe acute respiratory syndrome coronavirus 2 (SARS-CoV-2), termed coronavirus disease 2019 (COVID-19), has generated a notable challenge for diabetic patients. Overall, people with diabetes have a higher risk of developing different infectious diseases and demonstrate increased mortality. Type 2 diabetes mellitus (T2DM) is a significant risk factor for COVID-19 progression and its severity, poor prognosis, and increased mortality. How diabetes contributes to COVID-19 severity is unclear; however, it may be correlated with the effects of hyperglycemia on systemic inflammatory responses and immune system dysfunction. Using the envelope spike glycoprotein SARS-CoV-2, COVID-19 binds to angiotensin-converting enzyme 2 (ACE2) receptors, a key protein expressed in metabolic organs and tissues such as pancreatic islets. Therefore, it has been suggested that diabetic patients are more susceptible to severe SARS-CoV-2 infections, as glucose metabolism impairments complicate the pathophysiology of COVID-19 disease in these patients. In this review, we provide insight into the COVID-19 disease complications relevant to diabetes and try to focus on the present data and growing concepts surrounding SARS-CoV-2 infections in T2DM patients.

## 1. Introduction

The current severe acute respiratory syndrome-coronavirus-2 (SARS-COV-2) outbreak has become a global health threat and generated an emergency situation worldwide [1,2]. As of May 2021, over 163,738,674 cases and 3,384,750 deaths were reported across the world [3]. COVID-19 manifests as a mild disease in a significant number of infected patients; however, respiratory failure and pneumonia, as well as multi-organ dysfunction and septic shock, characterize patients with severe disease [2,4]. Patients’ pre-existing medical conditions and their immune system status play a pivotal role in the pathogenicity of the disease [5]. Individuals with underlying conditions, including hypertension, cardiovascular disease, chronic lung and renal disease, and diabetes mellitus (DM), are more likely to experience severe COVID-19 infection and its related mortality [4,6,7].

In general, coronaviruses (CoVs) primarily infect the upper respiratory tract and gastrointestinal tract. These viruses cause a range of symptoms from mild infections to severe manifestations including bronchitis, pneumonia, and renal dysfunction [4,8]. Since 2000, two deadly outbreaks in China (2002) and Saudi Arabia (2012) were caused by two highly pathogenic beta-CoVs—SARS-CoV and Middle East respiratory syndrome coronavirus (MERS-CoV), respectively [1]. These two infamous coronaviruses have been linked to fatal illnesses characterized by respiratory tract infections followed by bronchitis and pneumonia [4,9].

The SARS-CoV outbreak, which began in the Guangdong Province in China and spread to the other countries in Asia, North America, and Europe [8], caused deadly pneumonia with a mortality rate of 10% [4]. Old age and underlying conditions were the leading risk factors associated with SARS-CoV disease development and increased the mortality rates by 50% among these patients [8]. In addition, elderly people with comorbidities are more susceptible to be infected with MERS-CoV, one of the most dangerous viruses to human health [8,10]. Diabetes, kidney disease, heart conditions, underlying respiratory disease, and hypertension have been shown to be associated with severe or lethal MERS-CoV infections [10], as MERS-CoV patients with these afflictions have a mortality rate of 36% [4]. Although fatality rates of SARS-COV-2 are lower than SARS-COV and MERS-CoV [11,12], old age and (or accompanied by) these same underlying diseases are reported as significant predictors of mortality among COVID-19 patients [4,13].

It has been estimated that DM is the second most prevalent comorbidity in patients with severe COVID-19 infection after hypertension [13,14]. In regard to previous studies of respiratory tract infections, the presence of DM, particularly Type-2 Diabetes Mellitus (T2DM), is related to higher risk of acquiring infections with worse clinical outcomes and mortality [13,15,16]. Studies in Wuhan, China and Italy indicated that DM is common among COVID-19 patients [13]; however, the reported prevalence of DM among COVID-19 patients varies by region, age, and ethnicity [4,7]. Most of the studies have shown that DM is associated with disease severity, poor prognosis, and mortality among COVID-19 patients [7,17]. However, how DM contributes to the severity of COVID-19 is unclear; systemic inflammatory responses and impaired immune system functions might be correlated with hyperglycemia and insulin resistance in these patients [4,6,7]. Due to the elevated levels of pro-inflammatory cytokines in severe COVID-19 cases [18], and the pre-existing state of metabolic inflammation in T2DM, the combination of the SARS-CoV-2 infections and T2DM might complicate and prolong lung injuries [19].

Similar to the SARS-CoV, the spike protein of SARS-CoV-2 is optimized for binding to the human Angiotensin-Converting Enzyme 2 (ACE2) receptor. ACE2 is an ectoenzyme that is expressed in different human organs, including the lower respiratory tract, kidney, myocardium, pancreas, and gastrointestinal tract. SARS-CoV-2 mainly exploits ACE2 to enter human cells [12]. DM rodent models demonstrate ACE2 upregulation in the lung, heart, kidney, and pancreas cells [6]. Administration of angiotensin-converting enzyme (ACE) inhibitors, Angiotensin II type-I Receptor Blockers (ARBs), and thiazolidinediones (TZDs) to control hyperglycemia is related to increased expression of ACE2 receptor [20]. Exposure of the ACE2 receptor to SARS-CoV-2 causes cytopathic effects on the human airway epithelial cells in vitro [12,21]. Moreover, a higher expression of ACE2 in human lung tissue has been associatedwith DM and its related treatments, which might increase sensitivity to SARS-CoV-2 infections [22]. These findings support the assumption that DM may contribute to the severity and excess mortality of the COVID-19 disease [6]. In this review, we discuss current and evolving concepts relevant to DM, and how adipose health impacts the pathogenicity and complications of COVID-19. In addition, we give a brief overview of COVID-19 variants and vaccines.

## 2. SARS-CoV-2, Family and Structure

Human CoVs are enveloped single-strand RNA viruses first described in 1960 in association with the common cold [8]. More CoVs have been discovered since, including alpha-CoV, beta-CoV, gamma-CoV, and delta-CoV genera [1]. The pathophysiology of the novel coronavirus, COVID-19, is similar to that of two previous beta coronaviruses, SARS-CoV and MERS-CoV, though COVID-19 shows higher transmissibility and some discrete clinical features [1,23]. Phylogenetic analyses revealed that the novel CoV, SARS-COV-2, also belongs to the beta-CoV genera and is more closely related to bat-SL-CoV ZC45 and bat-SL-CoV ZXC21 than to human SARS-CoV and MERS-CoV [1,24,25].

CoVs have the largest known genome among RNA viruses, with the size ranging from 26–32 kb. CoV genomes encode 16 nonstructural proteins (nsp1–nsp16) through ORF 1a/b at the 5ʹ end. Other ORFs located at the 3ʹ end of the genomic RNA encode structural proteins including spike (S), envelope (E), membrane (M), nucleocapsid (N), and accessory proteins, which vary in number and location for each strain [8,24,26]. The genomic length of SARS-COV-2 ranges from 29.8 kb to 29.9 kb and mimics the characteristics of other CoV genomes [27]. The genome sequence alignment indicates that single nucleotide polymorphisms (snps) are more conserved among nonstructural proteins of different CoVs (58% identity), while structural proteins are more diverse (43% identity) to meet the host adaption requirements [24]. Structural proteins are essential for virus assembly, and S glycoproteins promote CoVs entry to the human cells [21]. The most abundant of CoVs structural proteins, the M protein, has three transmembrane domains and is critical for the intracellular formation of virus particles [24]. The packaging of the encapsulated genome and virus assembly is conducted via the E and N proteins [24,28]. 

CoV S proteins play a pivotal role in the pathogenesis of the virus and are the main targets of neutralizing antibodies [21,28]. The amino acid sequence of S proteins in SARS-CoV-2 is ~77% identical with the prior SARS-CoV S proteins, [29] and both of them are optimized to bind the human ACE2 receptors [21,30]. The SARS-CoV-2 S protein is a trimeric protein that contains two functional subunits; the S1 subunit is engaged at receptor binding, and the S2 subunit promotes viral and host cell membrane fusion [1,21]. A polybasic (furin) cleavage subunit is located at the S1–S2 boundary, which increases the viral infectivity. [11,30]. The binding affinity of the SARS-CoV-2 S protein for hACE2 is as strong as the SARS-CoV S protein, [21] and the increased length of this protein in SARS-CoV-2 is thought to correlate with the pathogenesis and disease severity [21,28]. Given the absence of specific antiviral therapy for CoVs [24], the S protein’s key role in determining virus virulence and tissue tropism has placed it in the spotlight for the development of drugs and vaccines to combat the SARS-COV-2 infection [1]. 

## 3. COVID Variants

Constant mutations or changes in the genetic code of viruses are a naturally occurring phenomena and lead to new variants with different characteristics [31,32]. These mutations are of particular concern because they may spread easier, cause more severe disease, or may evade the body’s immune response. This includes SARS-CoV-2, where genomic sequencing enabled researchers to identify virus variants and their characteristics [33]. To date, there are multiple COVID-19 variants circulating globally that spread more quickly than other variants (Table 1). Currently, multiple investigations to understand the severity and characteristics of these variants are underway.

## 4. Clinical Data

The primary symptoms of COVID-19 infection include fever, dry cough, and dyspnea triggered after an average of 5.2 days incubation [5,6,34]. Sputum production, myalgia, headache, pharyngeal pain, abdominal pain, diarrhea, hemoptysis, conjunctivitis, and lymphopenia are other clinical manifestations that are common among COVID-19 patients [1,5,35]. In general, the spectrum of clinical characteristics varies among COVID-19 patients and comprises asymptomatic carriers, mild cases, acute respiratory disease (ARD), and death [5,23]. Although the frequency of asymptomatic cases with COVID-19 is considerable (20–86%), clinical and pathological data of severe cases indicated massive alveolar injuries and pneumonia, which can result in Acute Respiratory Distress Syndrome (ARDS), multi-organ failure, septic shock, and death [5,23]. The period from the first symptom appearance to death ranges from 6–41 days (median 14.0 days) and may be decreased among patients > 70-years old depending on the status of their immune systems [5,36].

COVID-19 is highly contagious, and it has been reported that men with comorbidities are more susceptible to be affected by the virus [37]. Coexisting medical conditions such as hypertension, DM, and cardiovascular disease have been observed in nearly half of COVID-19 cases [1,37], and these conditions also increase the risk of severe manifestations and mortality [1,6]. Elevated levels of pro-inflammatory cytokines including tumor necrosis factor alpha (TNF-α), interleukin (IL)-1 and IL-6, as well as lymphopenia, are the most prevalent cytokine profile shifts in COVID-19 patients with severe or terminal disease [1,23,37]. High levels of alanine aminotransferase (ALT) or aspartate aminotransferase associated with liver dysfunction have been observed in COVID-19 patients [1,34]. Liver biopsies show moderate microvesicular steatosis and mild lobular activity related to liver damage caused by either SARS-CoV-2 infection or drug administration [23].

Damage to other organs, including to the heart and kidneys, has been reported in severe COVID-19 cases. It has been remarked that patients’original physiologic states and underlying diseases may influence the complication and treatment of COVID-19, especially in older patients [4,14]. Pancreatic injuries have been reported in hospitalized patients with COVID-19, which might be caused by the direct binding of SARS-COV-2 to the ACE2 receptors in pancreatic islets or indirectly as a result of ARDS and multi-organ dysfunction [35]. Furthermore, abnormally elevated levels of enzymes, including lactic dehydrogenase (LDH), α-hydroxybutyrate dehydrogenase (α-HBDH), ALT, and gamma-glutamyl transferase (GGT) have been associated with multi-organ failure, which is more serious in diabetic patients [38]. 

## 5. T2DM and Infectious Diseases

T2DM is a universal public health problem. In 2015, 415 million adults were diagnosed with T2DM globally, and every six seconds one person died from this devastating disorder. It is estimated that 642 million people will suffer from T2DM by 2040 [39]. T2DM is a chronic and progressive metabolic disease that is linked to several serious health problems, including cardiovascular disease, blindness, kidney failure, cognitive decline, and premature death [40]. T2DM is defined by prolonged hyperglycaemia, insulin resistance, and relative insulin deficiency, and comprises about 85–95% of the global prevalence of DM [41,42]. A complex interaction of genetic and environmental factors predisposes people to insulin insensitivity and T2DM development [42,43]. Due to a decreased T-cell-mediated responses [44] and impaired neutrophil function [45], immune system function is compromised in diabetics, and these individuals are more susceptible to infectious diseases [46]. T2DM patients are more susceptible to infections in the lower respiratory tract, urinary tract, skin and mucous membranes [47]. The risk of infection-related mortality is also ten times higher in DM patients compared to the general population [48]. T2DM is one of the most common comorbid conditions in SARS and MERS-CoV coronavirus infections. Investigating the relationship between T2DM and COVID-19 will have a positive clinical consequence and improve the management of diabetic patients affected by COVID-19 [19].

## 6. T2DM and COVID-19 Infection

In December 2019, a group of patients with an unidentified pneumonia (viral infection) had been recognized in Wuhan, China. The viral infection was caused by the novel coronavirus, SARS-CoV-2 [49]. Reports from the Centers for Disease Control and Prevention indicated that T2DM patients have increased risk of mortality when they are exposed to COVID-19 [50]. It was shown that DM was one of the most distinctive comorbidities in 32 non-survivors out of 52 intensive care unit patients with COVID-19 [51]. Another study revealed that 12% of 140 patients infected with COVID-19 that were admitted to a hospital were diabetics [52]. In yet another study, diabetics represented 16.2% of patients infected with COVID-19 who had metabolic diseases [53]. 

In Hong Kong, China, the COVID-19-related fatality rates of elderly patients (age 75 or over) with T2DM were higher than the fatality rates in elderly patients suffering from cardiovascular diseases or cancer [54]. The same trend was reported for SARS-CoV in 2002 and MERS-CoV in 2012 [55,56].

## 7. COVID-19 Mechanism of Action in T2DM

**Figure 1:**
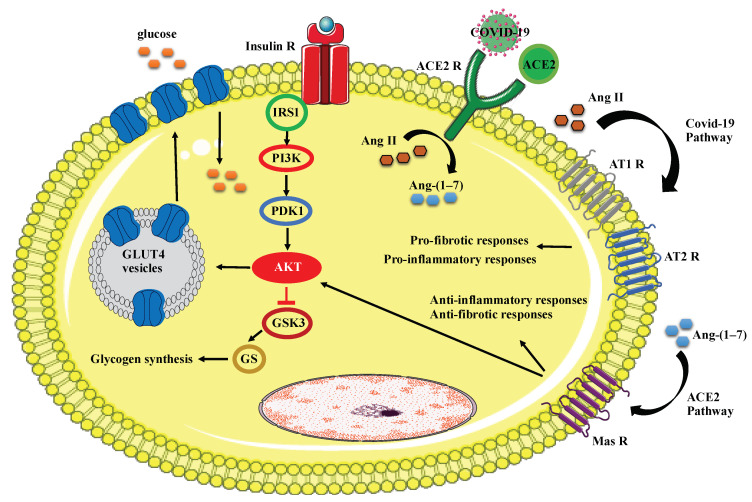
COVID-19 mechanism of action. Phosphorylation of Akt leads to the translocation of Glut4 to the cell membrane, which facilitates glucose uptake. ACE2 induces anti-fibrotic and anti-inflammatory responses and activates Akt through the Mas receptor. COVID-19 activates AT1 and AT2 receptors, which results in the generation of pro-fibrotic and pro-inflammatory responses in the cell. Insulin R: insulin receptor, IRS1: Insulin Receptor Substrate 1, PI3K: Phosphoinositide 3-kinase, PDK1: Phosphoinositide-dependent kinase-1, GSK3: Glycogen synthase kinase, GS: Glycogen synthase, ACE2 R: ACE 2 receptor, Mas R: Mas receptor.

Using the envelope S glycoprotein, which is found on the surface of the virus, COVID-19 enters human cells by binding to the ACE2 receptors on human cell surfaces (Figure 1) [57]. In the pulmonary system, ACE2 (mentioned above) is the key regulatory point of the angiotensin system responsible for degrading angiotensin II (Ang II) into Ang-(1–7) [58]. Ang-(1–7) acts on the Mas receptor pathway, which leads to anti-inflammatory and anti-fibrotic responses and, therefore, helps the recovery of infected patients with COVID-19 [57]. Ang-(1–7) also stimulates the insulin signalling pathway through the Mas receptor and ameliorates the depriving effects of Ang II. Ang-(1–7) mediates insulin actions in metabolic organs through Akt activation [59]. It is well defined that the phosphorylation of Akt is involved in the activation of downstream pathways of glucose metabolism. These include SHP-2, ERK1/2, PRAS40, and GSK-3beta, which facilitate the translocation of the Glut-4 glucose transporter from the cytoplasm to the cell membrane to take up glucose and subsequently reduce blood sugar levels [60,61,62]. When COVID-19 binds to ACE2 receptors, ACE2 activities become inhibited. Therefore, Ang II, which is the key molecule in the renin–angiotensin system (RAS) [63], is forced to act via the angiotensin 1 (AT1) and angiotensin 2 (AT2) receptors to exert pro-inflammatory responses [57]. In more severe COVID-19 infections, there is a lack of balance in the activation of ACE2 versus AT1/2 receptors involved in these signalling pathways, which results in the increased activation of AT1 and AT2 receptors and consequently leads to insulin resistance and T2DM conditions [19]. 

It has been reported that SARS-CoV (1–2), binds to ACE2 receptors in pancreatic cells and destroys islets, causing a decrease in the production and release of the insulin hormone. Even in non-diabetic individuals affected by SARS-CoV, the coronavirus might enter pancreatic islets expressing ACE2 receptors, leading to acute β-cell dysfunction and transient T2DM. For example, it has been shown that more than 50% of the patients affected by SARS-CoV, without any history of T2DM or steroid treatments, became diabetic during hospitalization. Then, three years after the recovery from the SARS-CoV infection, the number of diabetic individuals decreased from 50% to 5% [64]. In mice, the activity levels of ACE2 in pancreatic islets were increased in DM patients, suggesting that diabetics are more susceptible to coronavirus effects. In addition, this study demonstrated that T2DM contributes to multi-organ failure in SARS-CoV infections as it induces the ACEs expression in the other cell types, including lung epithelial cells, hepatic and cardiac cells [65]. 

## 8. Diabetes, COVID-19, and Immune Response

How DM increases the severity of COVID-19 has yet to be fully undertsood. As noted in other CoV infections, the mere binding of the virus to ACE2 does not cause severe lung injuries. Pulmonary damage, which manifests following the SARS-COV-2 infection, might become more complicated due to the dysregulation of adaptive and innate immune system responses [6,7]. A cohort study of 452 patients in Wuhan, China, indicated that immune system malfunction occurred during COVID-19 disease and at least one underlying disease was reported in 44% of this cohort. [37]. In addition, higher concentrations of granulocyte colony-stimulating factor (GCSF), IFN-γ-inducible protein 10 (IP10), monocyte chemoattractant protein-1 (MCP1), macrophage inflammatory protein 1-A (MIP1A), and TNFα cytokines were observed in this cohort of COVID-19 patients admitted to the intensive care unit, suggesting that a cytokine storm was associated with COVID-19 severity. [66]. Likewise, it has been demonstrated that there is a critical relationship between cytokine storms and morbidity in patients with SARS-CoV and MERS-CoV [67]. Furthermore, elevated levels of IL-6, a hallmark of severe MERS-CoV infection, is common in COVID-19 patients with respiratory failure and ARDS. [68]. Another key feature of immune system dysfunction that is related to COVID-19 severity is lymphopenia [37,68,69]. Indeed, lower T cell function causes innate immune system dysregulation and a cytokine storm, which leads to widespread inflammation in the lungs and subsequent respiratory failure, ARDS, and multi-organ dysfunction that is related to COVID-19 severity [6,69,70]. Diabetic patients’ pre-existing, pro-inflammatory state might facilitate severe COVID-19 infections [71]. Immune system impairment is associated with T2DM and abnormal secretion of pro-inflammatory cytokines, particularly TNFα and IFN in COVID-19 patients. [7,72]. Furthermore, DM is associated with increased C-reactive protein (CRP), fibrinogen, and D-dimer that can lead to the hypercoagulation state observed in COVID-19 patients with DM. [14,38,72].

In general, patients with diabetes have an increased predisposition to infectious diseases and related complications and mortality. DM is associated with COVID-19 immune response complications; however, how it increases the disease severity is unclear, and further investigation is critical to reveal these mechanisms.

## 9. Multi-Omics View of COVID-19 and T2DM

**Figure 2:**
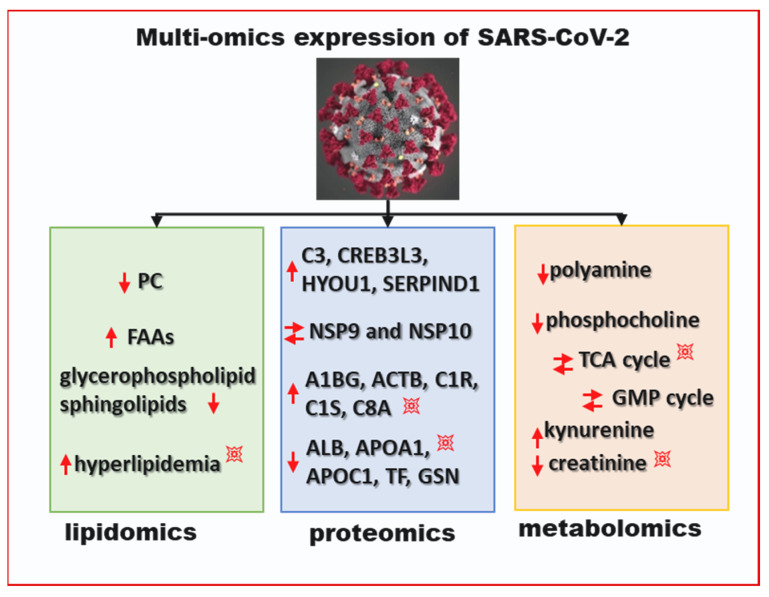
Multi-omics expression of SARS-CoV-2. The blast symbol (💥) designates the accentuated regulation of the process in T2DM. The down arrow indicates downregulation and the up arrow indicates upregulation; two parallel arrows symbolize differential expression compared to control after SARS-CoV-2 infection. PC: phosphatidylcholine; FAAs: free fatty acids; GMP: guanosine monophosphate; TCA: citric acid cycle.

COVID-19 patients show heterogeneity in their manifestation of symptoms, which are primarily based on the individuals’ health. T2DM individuals with a compromised immune defense have a much higher risk of symptomatic COVID-19 and mortality. For instance, a meta-analysis of >40,000 patients demonstrated that COVID-19 patients with T2DM had a four times higher fatality rate [73]. Although a few studies identified impaired T cell function or the presence of higher inflammatory factors as potential reasons for the increased risk of COVID-19 infection-related hospitalisation in diabetics, the causation is still unclear. It is also not clear if DM itself is a risk factor or its association with the other cardio-metabolic diseases such as obesity, dyslipidemia, and hypertension, drive the heightened risk for COVID-19 mortality in T2DM patients that contract the virus. A multi-omics study with COVID-19 serum uncovered dysregulation of several proteins and metabolites between the samples from relatively healthy patients and those from patients with severe disease symptoms [74]. It was found that the cellular macrophage proteins APOA1, APOA2, and APOH were downregulated with severity of COVID-19 disease, whereas acute phase proteins (APP) SAA1, SAA2, SERPINA3, C5, C6, and C8 were upregulated. The protein C5a is upregulated in patients with ARDS and lung injury and is not directly associated with the T2DM [75]. However, the upregulation of the metabolites glucose, glucuronate, and bilirubin with COVID-19 patients indicate that the liver damage typically co-occurs in DM patients [76]. For the T2DM patients who already have higher reactive oxygen species (ROS) levels, ROS levels further increase and lead to the accelerated cellular damage associated with COVID-19 infection [77]. Alteration of the tricarboylic acid cycle was observed through serum metabolomics analysis of COVD-19 patients. Malic acid andglycerol 3-phosphate were found to be significantly different between healthy and COVID-19 infected patients. These metabolites are also involved in the hepatic distress that is common in T2DM patients [78]. A lipidomic study identified alterations in phosphatidylcholine and glycerophospholipids, and related hyperlipidemia in COVID-19 cases [78,79]. T2DM patients suffer a reduction in high density lipoprotein cholesterol that is accentuated by SARS-CoV-2 infection. The multi-omics analyses determined the lipid, protein, and metabolite alterations that occur during SARS-CoV-2 infection and are presented in Figure 2. As discussed earlier, many of the observed pathways that are differentially expressed in SARS-CoV-2 patients are already altered in DM patients. Upon infection, these processes are accentuated and lead to a rapid reduction in T2DM patients’ conditions and increase mortality.

## 10. Important Recommendations for COVID-19 Infected Patients with T2DM

According to the severity of COVID-19 infection in diabetic patients, treatment policies, including blood glucose target levels, are different. Blood glucose monitoring, dynamic assessments, and timely adjustments should be reinforced to promote the safety and early recovery of patients [80]. Sardu et al. demonstrated that the optimal glucose control is associated with a significant reduction in inflammatory cytokines and COVID-19 disease severity. They showed that insulin infusion might be useful in obtaining glycemic targets and reducing mortality in diabetic patients with COVID-19. Achieving glycemic targets would enable the combination of an antidiabetic, anti-inflammatory, and antiviral effects of the SARS-CoV-2 infection treatment in these patients [81].

While the expression of ACE2 is increased in the early stages of DM, its mRNA and protein expressions decrease in older streptozotocin-induced diabetic rats [58]. ACE2 expression is increased in diabetic patients treated with ACE inhibitors and ARBs [82]. Accordingly, the increased expression of ACE2 with ACE2-stimulating drugs likely facilitates the virus’s entrance and consequent cell infection, and enhances the risk of developing severe and fatal COVID-19 [20]. In another study, it was mentioned that early treatment of COVID-19 infection with ARBs, for example, losartan or telmisartan, or recombinant ACE2, might be useful to increase the ACE2 activity and Mas system to promote signaling pathways mediated by angiotensin receptors, including the insulin signaling pathway [19]. Glucagon-like peptide-1 (GLP1) agonists protect cells from the coronavirus entrance by competitively binding to ACE2 [19]. Camustat, a synthetic protease inhibitor, blocks the TMPRSS2, a type II transmembrane serine protease, which is required to prime viral entry into the cells via ACE2 [19].

Even though previous animal investigation showed that ACE inhibitors and ARBs increase ACE2 activity, neither ACE2 mRNA expression changes in rat heart cells [83] nor have plasma ACE2 activity changes have been found in the presence of either ACE inhibitors or ARBs in humans [84]. However, current evidence on COVID-19 and ACE inhibitors or ARB medication is controversial, and the correlation of ACE2, DM, hypertension, and severity of COVID-19 cannot be as simple as it seems [72]. Investigation on the ACE2 polymorphism associated with T2DM and hypertension and its link with increased risk of SARS-CoV-2 infection would be beneficial in COVID-19 therapy. Fang and colleagues have suggested that antihypertensive calcium channel blockers would be useful for COVID-19 patients with cardiac diseases, hypertension, or DM since there is no evidence of ACE2 expression induction with these agents [20]. However, there is not adequate evidence to confirm the risk or beneficial effects of ABRs, ACE inhibitors, thiazolidinediones (TZDs), or GLP-1 agonists in COVID-19 patients (6). Hence, the American College of Cardiology, the American Heart Association, the American Society of Hypertension, and the European Society of Cardiology have recommend that using ACE inhibitor medications in patients with comorbidities should not be interrupted because of SARS-CoV-2 infection [6,13,85].

Due to the high amounts inflammatory cytokines, corticosteroids were used frequently for treatment of severe cases of MERS-CoV and SARS-CoV infections. However, it has been suggested that corticosteroids might inhibit immunity and SARS-CoV-2 clearance. Therefore, the World Health Organization’s interim guidance on clinical management of COVID-19 raises concerns about corticosteroid usage outside of clinical trials [4]. The antimalarial drug hydroxychloroquine (HCQ) has also been reported as a potential antiviral drug. Although the efficiency and safety of HCQ for COVID-19 is challenging, its immunomodulant and anti-inflammatory effects and its positive role in glucose homeostasis regulation have highlighted it as a potential treatment for COVID-19 infected patients with DM [4,86]. Although hyperglycemia is the main concern in diabetic patients infected with SARS-CoV-2, there is no specific treatment to manage these patients [4,80]. Further investigation of optimal glucose control strategies to prevent disease severity, and of the presence of comorbidities and their association with demographic features are critical in this context.

## 11. Adipose Health and SARS-CoV-2 Susceptibility

With the SARS-CoV-2 pandemic is still actively impacting international communities, recent investigations have tried to decipher why the virus differentially impacts individuals. As over 1.9 billion individuals are overweight worldwide [87], how SARS-CoV-2 affects obese individuals has become a focus of investigation. Reviews of SARS-CoV-2 outcomes reveal that obesity is a largely unfavorable co-morbidity, as obesity worsens the infection itself, increases hospitalizations, and increases mortality [88].

SARS-CoV-2 exploits obese white adipose tissue in order to propagate. SARS-CoV-2 uses a viral spike protein to enter target cells via binding to human ACE2 and dipeptidyl peptidase 4 (DDP4) to enter host cells. ACE2 is highly expressed in white adipose tissue (AT), and is more highly expressed in visceral compared to subcutaneous adipose depots [89,90]. ACE2 expression is particularly upregulated in adipocytes from metabolic unhealthy obese (MUO) individuals and in the heart, lung, and kidney tissue of DM mice. Identified as a novel adipokine that is secreted from adipocytes, DPP4 plays roles in glucose homeostasis and inflammation in white adipose [91,92]. Obese individuals demonstrate increased adipose DPP4 secretion compared to lean individuals, and the inhibition of DPP4 in obese mice abated fibrosis in white adipose [93]. The high expression levels of ACE2 and DPP4 in obese AT facilitates the circulation of SARS-CoV-2 and its downstream cytokine storm.

Increased inflammation in visceral adipose with obesity makes MUO individuals more susceptible to severe SARS-CoV-2 infection. Visceral AT deposition is accompanied by pro-inflammatory macrophage accumulation as well as adipocyte hypertrophy, mitochondrial dysfunction, and increased ROS, which incite further pro-inflammatory macrophage recruitment. The tissue dysfunction leads to increased secretion of cytokines, including IL-6, TNF-α, and IL-1β, from both adipocytes and macrophages. This increase in pro-inflammatory cytokine secretion was thought to couple visceral obesity and influenza-related respiratory complications, and is now hypothesized to play a role in the cytokine storm observed in patients with severe SARS-CoV-2 [94]. This information indicates that, while all obese individuals are at risk for their white adipose acting as a reservoir for SARS-CoV-2, MUO individuals may be at an increased risk of severe consequences, given their visceral adipose accumulation and its corresponding pro-inflammatory macrophage accumulation and cytokine secretion.

## 12. Vaccines and DM

Two of the most well-known CoVs known prior to SARS-CoV-2 were SARS and MERS [30,95]. There are no approved vaccines for SARS or MERS. Preceding work to develop vaccines against these targets established the required knowledge about the structural biology and functions of CoVs, and enabled the researchers worldwide to accelerate the development of COVID-19 vaccines and provide acquired immunity to popoulations at risk [96]. Prior to the COVID-19 pandemic, the average time to develop a vaccine against viral infections was around seven years [97]. The urgency to develop a COVID-19 vaccine led to unprecedented schedules that curtailed the standard vaccine development timeline [98]. The rapid development of COVID-19 vaccines has surpassed several unique challenges (safety, efficacy, dose regimen, stability, and storage characteristics) and began at the time of isolation and identification of the SARS-CoV-2 viral genetic sequence [99]. Furthermore, challenges such as national lockdowns and physical distancing directly increased the concerns over the safety of the vaccines.

According to the World Health Organization (WHO, as of 21 May 2021), there are around 284 vaccines in various developmental stages. Of the 284 candidates, 183 are in pre-clinical development and 101 are in the clinical development phases (Table 2) [96,97,100]. In phase III clinical trials, several vaccines worldwide have demonstrated efficacy of higher than 95% in prevention of COVID-19 infection. To date, at least ten vaccines have been authorized for public use (full or emergency) and as of 21 May 2021, over 700 million doses (counted as a single dose, and may not equal the total number of people vaccinated) have been administered worldwide (Table 3) [100]. To date, the Oxford–AstraZeneca/Covishield vaccine (AZD1222), Pfizer–BioNtech, Moderna vaccine, Johnson and Johnson (Ad26.COV2.S), and Sinopharm (BBIBP-CorV) vaccines are approved and have recieved recommendations for full use by the WHO Strategic Advisory Group of Experts on Immunization [101].

The WHO has recommended that people with comorbidities that have been identified as being at an increased risk of severe COVID-19, including obesity, cardiovascular disease, respiratory disease, and DM, receive COVID-19 vaccines [99]. However, researchers have pointed out an important caveat of elevated levels of blood glucose for diabetic individuals post-vaccine administration due to higher energy consumption in response to immune functions [102]. In the United States, the Pfizer–BioNtech trial included 3,150 DM people (8.4% of trial participants) [103] and the Moderna trial included 2,858 people with type 1, type 2, and gestational DM (9.4% of trial participants) [104]. The Oxford–AstraZeneca vaccine trial had > 5% [105] and the Sputnik V trial enrolled 3687 people (24.7%) [106] with diabetic and cardiovascular commodities. All of these above-mentioned vaccine clinical trials demonstrated that the vaccines were safe, highly efficacious and produced immune responses. Nonetheless, there are still a lot of questions about (a) reinfections, (b) long-term immunity, (c) the rate protection against COVID-19, and its variants in DM compared to healthy populations, (d) drug interactions with the vaccine, (e) reasons for delaying 2nd dose, mixing two different vaccines, and no commensus on the vaccine dose schedule between two shots between countries, and (f) safety of vaccines in immuno-compromised individuals with DM. Moreover, there are no approved vaccines for individuals below 12 years of age and asymptomatic infections, which requires urgent attention.

**Table 3:** List of key vaccines authorized for emergency use, approved for full use, or pending worldwide. Note: Dosing schedule mentioned in the table is based on healthy/non-immuno-comprosmised peoeple, but can be different/delay in 2nd dose depending on age/health status and recommendations from health authorities in individual countries.

Vaccine/Company/Candidate	Country	Platform ^#^	Efficacy *	Age Group	Doses ^+^	Storage	Status	Ref
Pfizer–BioNTech vaccine	United States, Germany	RNA	95%	12yrs and older	2 doses, 21 days apart(USA), 6 week(EU/UK)	−80 °C	Full use	[103]
Moderna vaccine	United States	RNA	94%	12yrs and older	2 doses, 28 days apart(US), 6 week apart (UK/EU)	−20 °C	Full use	[104]
Oxford–AstraZeneca vaccine (AZD1222)	United Kingdom	VVnr	Overall: 70%, Dose-based: 62% to 90%	18 yrs and older	2 doses, 12 week apart	2–8 °C	Full use	[105]
BBV152 (Covaxin)	India	IV	Preliminary efficacy estimation by end of February 2021	18 yrs and older	2 doses, 14 days apart	2–8 °C	Emergency use	[107]
Sputnik V vaccine	Russia	VVnr	91.6%	18 yrs and older	2 doses, 21 days apart	2–8 °C	Full use	[106]
EpiVacCorona	Russia	PS	100% in early trials	18 yrs and older	2 doses, 21 days apart	2–8 °C	Full use	
CoronaVac	China	IV	50%–91% (Turkey, Brazilian and Indonesian cohorts)	18 yrs and older	2 doses, 21 days apart	2–8 °C	Full use	[108]
BBIBP-CorV	China	IV	79.34%	18 yrs and older	2 doses, 14 days apart	2–8 °C	Full use	[109]
Ad5-nCoV (Convidicea)	China	VVnr	66%	18 yrs and older	1 dose	2–8 °C	Emergency use	[110]
Ad26.COV2.S or JNJ-78436735	Netherlands	VVnr	85% against severe COVID	18 yrs and older	1 dose	2–8 °C	Full use	[111]

**^#^** Refer Table 1, * Symptomatic infection and severe disease, **^+^** Intramuscular route.

## 13. Conclusions

Whether people with DM have a greater risk of contracting COVID-19 infections is currently unknown. However, increased pulmonary damage, which manifests following SARS-COV-2 infection, is thought to occur in T2DM patients. How DM increases the severity of COVID-19 is challenging to summarise. The increased severity might be associated with chronic inflammation and the immune system impairment present in patients with T2DM. Like any form of infectious disease, the hyperglycemic state is an important prognostic factor in diabetic patients exposed to SARS-CoV-2. Elevated levels of pro-inflammatory cytokines and the cytokine storm observed in severe cases of COVID-19, as well as the metabolic inflammation that presents in T2DM, might participate in complicated and prolonged lung injuries in COVID-19 patients. Elevated blood glucose may itself cause an inflammatory response and multi-organ failure leading to severe COVID-19 disease.

Studies about other CoVs suggest that SARS-CoV-2′s interaction with the ACE2 receptor may not cause severe disease manifestations. There might be a crucial correlation between immune system dysfunction, impaired inflammatory responses, metabolic abnormalities, and COVID-19 severity and mortality.

Nevertheless, the administration of ACE and ARB inhibitors to treat diabetes in COVID-19 patients is controversial. It has been demonstrated that optimal blood glucose control may itself be associated with a significant reduction in inflammatory cytokines and COVID-19 disease severity. It is suggested that combined antidiabetic, anti-inflammatory, and antiviral approaches would be an efficient strategy to combat SARS-CoV-2 infection in diabetic patients.

## Figures and Tables

**Table 1:** List of important SARS-CoV-2 variants and characteristics.

Variant	Location	Identified	Charactistics
B.1.1.7	United Kingdom	August/September 2020	Highly contagious with increased risk of death
B.1.351	South Africa	October 2020	A few similar mutations to B.1.1.7
P.1	Brazil	January 2021	Additional mutations that may subvert by antibodies recognitions
B.1.617	India	February 2021	Highly transmissible and capactity to reduce the post-vaccination sera

**Table 2:** Number of COVID-19 vaccine candidates and platforms in the late stage pre-clinical and clinical phases.

Platform		Clinical	Preclinical
PS	Protein subunit	31	70
VVnr	Viral Vector (non-replicating)	16	21
DNA	DNA	10	16
IV	Inactivated Virus	16	9
RNA	RNA	16	24
VVr	Viral Vector (replicating)	5	19
VLP	Virus Like Particle	5	18
VVr + APC	VVr + Antigen Presenting Cell	2	-
LAV	Live Attenuated Virus	2	2
VVnr + APC	VVnr + Antigen Presenting Cell	1	-
LABV	Live attenuated bacterial vector	-	2
BVr	Bacterial vector (Replicating)	-	1

## Data Availability

Not applicable.
